# Resection of an Extraovarian Noniuteinized Thecoma in a 14-Year-Old Girl

**DOI:** 10.1055/s-0042-1742712

**Published:** 2022-04-19

**Authors:** Ophelia Aubert, Robin Wachowiak, Christian Roth, Anne K. Höhn, Martin Lacher, Steffi Mayer

**Affiliations:** 1 Department of Pediatric Surgery, University Hospital Leipzig, Leipzig, Sachsen, Germany; 2 Division of Gynecologic, Breast and Perinatal Pathology, Institute of Pathology, University Hospital Leipzig, Leipzig, Sachsen, Germany; 3 Department of Pediatric Radiology, University Hospital Leipzig, Leipzig, Sachsen, Germany

**Keywords:** ovarian neoplasm, extraovarian thecoma, adolescent

## Abstract

Thecomas are rare benign sex cord-stromal tumors that account for less than 1% of all ovarian tumors. They usually affect postmenopausal women and become symptomatic with abnormal bleeding. In adolescents, less than 10 cases have been reported so far, mainly with symptoms of hormonal disbalance. Extraovarian thecomas represent an even rarer entity, with only two cases described so far, none of them in the pediatric population.

We report the case of a 14-year-old girl who presented with sudden-onset abdominal pain, dysuria, and fever, as well as highly elevated serum inflammation parameters. Ultrasound and magnetic resonance imaging (MRI) revealed a large, inhomogeneous pelvic mass (16 cm × 9 cm × 13 cm) with indistinct margins, suggestive of an infiltrative malignant teratoma or sarcoma. Laparoscopy confirmed a large mass of unknown origin. In contrast to the infiltrative character seen on preoperative MRI, the tumor could be easily exteriorized and resected after conversion to laparotomy. Ovaries, fallopian tubes, and uterus remained unaffected. Histopathology revealed a benign nonluteinized thecoma. The postoperative course and 19-month follow-up were uneventful.

## Introduction

Thecomas are benign sex cord-stromal tumors that account for less than 1% of all ovarian tumors, and typically affect postmenopausal women. 1 Extraovarian thecomas are an even rarer entity, and only two cases have been reported so far. These tumors are composed of lipid-containing cells resembling those of the theca interna. Thecomas are usually estrogen-producing, therefore most postmenopausal women present with abnormal uterine bleeding. 2 To date, less than 10 cases have been reported in children and adolescents. These patients have symptoms of hormonal disbalance like precocious puberty or androgenic manifestations. 3 4 5 6 7 Here, we present the rare case of a large extraovarian thecoma in a 14-year-old girl presenting with acute abdominal pain and highly elevated inflammatory parameters.

## Case Report

**Fig. 1:**
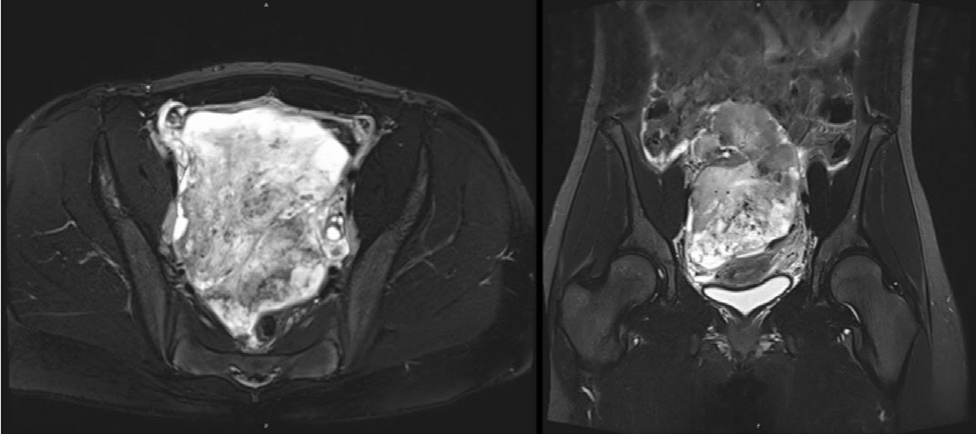
Preoperative MRI scan (T2-weighted sequence) revealed a large pelvic mass of unknown origin. MRI, magnetic resonance imaging.

A 14-year-old healthy girl presented with a 1-day history of sudden-onset abdominal pain in the left lower quadrant, dysuria, and menstrual bleeding. She had attained menarche at 11 years and had had regular menstrual cycles so far. Physical examination revealed normal sexual maturation (Tanner's stages B4 and P4) without signs of hormonal imbalance. She showed tenderness of the left middle abdomen but no palpable mass and subtle pain on percussion of the left kidney. The patient rapidly deteriorated with pronounced abdominal pain, vomiting, and fever. Ultrasound and magnetic resonance imaging (MRI) scan depicted a 16 cm × 9 cm × 13 cm mass adjacent to the ovaries, with inhomogeneous contrast enhancement and irregular margins in the greater and lesser pelvis, suggestive of a poorly differentiated teratoma or sarcoma ( Fig. 1 ). There was no ascites.

Laboratory testing showed elevated leucocytes (13,900 g/L), C-reactive protein (235.05 mg/L), cancer antigen 125 (CA; 125; 157.0 U/L; normal < 35 U/mL), as well as dehydroepiandrosterone sulfate (DHEAS; 7.76 µmol/L; normal: 0.47–5.79 µmol/L). Levels of estradiol, β-human-chorionic-gonadotropin, α-fetoprotein, carbohydrate antigen 19–9, and carcionembryonic antigen were normal.

**Fig. 2:**
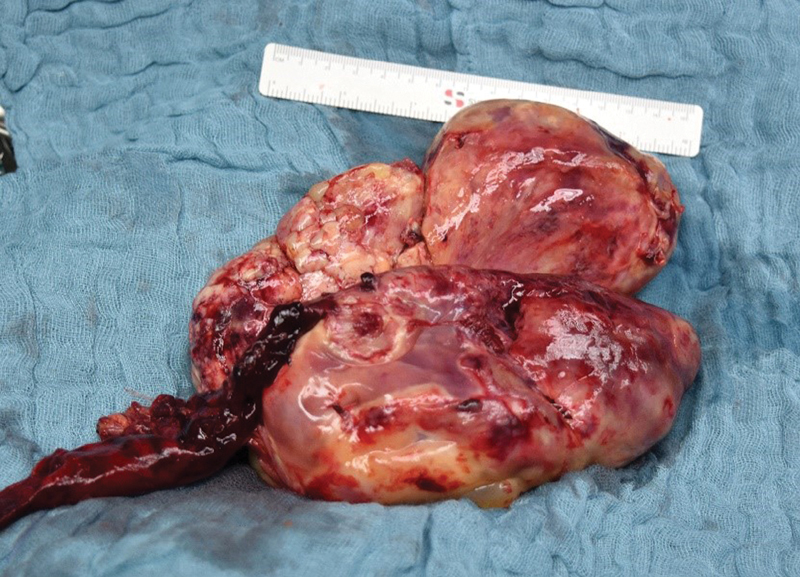
Resected thecoma (17 cm × 14.5 cm × 7.5 cm; 710 g) with adherent greater omentum.

Laparoscopy was performed the next day, exposing a tumor covered by greater omentum and with a punctual adhesion to the right ovary. Due to the tumor size and its unknown origin, the operation was converted to a median lower abdominal laparotomy. Ovaries, fallopian tubes, and uterus were considered normal. Unexpectedly, the tumor did not infiltrate any surrounding structures and could be exteriorized easily. An en bloc resection was performed preserving the internal genitalia ( Fig. 2 ). Furthermore, a small amount of peritoneal fluid was harvested. Intraoperative frozen section of an intraabdominal lymph node did not show any signs of malignancy but was consistent with a mesenchymal lesion.

**Fig. 3:**
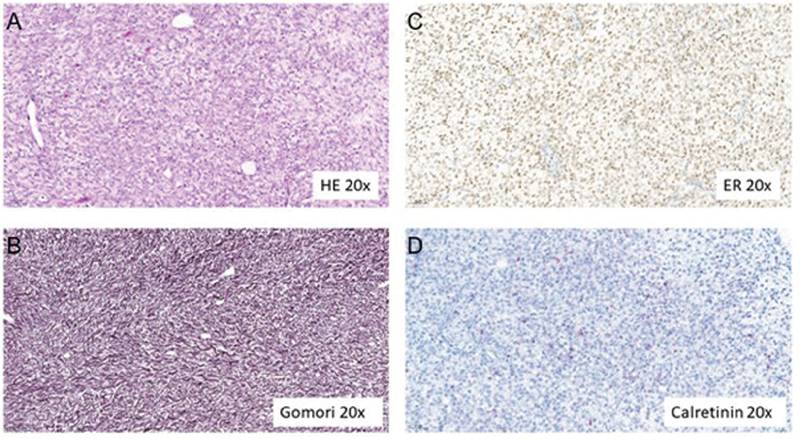
Staining and immunohistochemistry in thecoma tissue: hematoxylin and eosin (HE) staining ( **A** ), Gomori's methenamine silver (Gomori) staining ( **B** ), estrogen receptor (ER) immunohistochemistry ( **C** ), and calretinin immunohistochemistry ( **D** ) under magnification (×20).

Histopathologic examination of the tumor showed cystic and solid areas with residues of necrosis and hemorrhage. Microscopically, fibroblast-prolific tissue and uniform cells without any atypia were seen. Cluster of lutein cells were not observed. A dense reticulin fiber network was revealed by Gomori's silver stain. Immunohistochemistry confirmed reactivity to markers such as calretinin and CD99, but also to the steroid hormone receptor estrogen, consistent with a benign sex cord-stromal tumor, specifically a theca cell tumor ( Fig. 3 ). Peritoneal fluid indicated a florid infection and no signs of malignancy.

The postoperative course was uneventful, and the patient was discharged home on day 5. Considering the benign nature of the tumor, no adjuvant treatment but quarterly follow-up was established. By the time of her first follow-up examination 2 months after surgery, the girl had resumed her usual activities with no abdominal discomfort, CA 125 levels were normal, and ultrasound showed no sign of tumor recurrence. At the latest follow-up 19 months postoperatively, she presented in good general condition and tumor free.

## Discussion

Thecomas are a rarity in the pediatric population and only a few cases have been reported over the last decades. Likewise, in 20 years, only two out of 72 patients with ovarian sex cord-stromal tumors from the Kiel Pediatric Tumor Registry, a reference-database for pediatric tumors in Germany, were diagnosed with thecomas. 8

Extraovarian thecomas are an extraordinary rarity. Only two cases, both in adults and both originating from the broad ligament, have been described so far. 9 10 In our patient, the organ of origin could not be determined. The ovaries were considered normal and only minimal adhesions of the tumor to the right ovarian were observed. These might be explained by the ongoing strong peritoneal inflammation rather than the origin of the tumor. To our knowledge, this represents the first case of an extraovarian thecoma reported in an adolescent.

Surgery is the treatment of choice for these tumors. Radical bilateral salpingo-oophorectomy with total hysterectomy is recommended in post- or perimenopausal women. 11 This is due to the associated risk of endometrial adenocarcinoma of approximately 20% which is caused by ongoing estrogen secretion from theca cells. 12 However, in cases where preservation of fertility is important, unilateral salpingo-oophorectomy, or organ-preserving tumorectomy should be attempted. 13 The benign nature of thecomas and the low recurrence rate of 2% for fibroma/fibrothecoma further justify an organ-sparing approach from an oncological point of view. 14 These procedures can be performed by laparotomy or by laparoscopy. 13 15 In contrast to what we expected based on preoperative MRI scans, which suggested a malignant and invasive tumor, the tumor was not infiltrating surrounding tissues. Therefore, complete and ovary-sparing resection could be performed.

In the scarce literature available on infants and toddlers, signs of hormonal disbalance like precocious puberty, feminization, and vaginal bleeding are typical for thecomas. 7 In adolescents, only eight cases of thecomas have been reported so far. 3 4 5 6 8 16 17 Four patients presented with slowly progressing androgenic manifestations such as hirsutism and hoarseness and two with Meigs' syndrome. 3 4 5 6 16 17 Although DHEAS levels in our patient were elevated, the girl showed no sign of virilization or hormonal disbalance.

Abdominal pain has only been described for luteinized thecomas in young fertile women. This uncommon thecoma subtype, characterized by well circumscribed clusters of luteinized appearing cells causing abnormal proliferation of fibroblasts, is associated with sclerosing peritonitis. 18 Our patient also presented with acute abdominal pain, although luteinized cell clusters could not be detected. However, moderately elevated CA 125, high C-reactive protein (CRP) levels, and inflammation in peritoneal fluid are suggestive of acute peritonitis and may explain her complaints. 19 Thus, nonluteinized thecomas may also be able to induce a strong peritoneal inflammation with acute abdominal pain in fertile adolescents which has not been reported before.

## Conclusion

We report the first case of an extraovarian, nonluteinized thecoma of unknown origin in a 14-year-old girl, presenting with acute abdominal pain. Despite the expectation of an infiltrative character on MRI, the tumor could be easily resected via laparotomy.

## Notes

New Insights and the Importance for the Pediatric Surgeon We report the first case of an extraovarian thecoma of unknown origin in an adolescent girl. The atypical presentation with acute abdominal pain, as well as the unexpected easy en bloc resection of the tumor that stands in stark contrast with the preoperative imaging suggestive of an infiltrative and malignant tumor, further make this case remarkable and worthy of publication.
